# Noses and knees

**DOI:** 10.1038/s43856-021-00035-x

**Published:** 2021-09-22

**Authors:** Ben Abbott

**Affiliations:** Communications Medicine, https://www.nature.com/commsmed

## Abstract

Regenerative medicine may offer strategies to alleviate the debilitating symptoms of osteoarthritis and delay the time to joint replacement. In a study now published in *Science Translational Medicine*, Acevedo Rua and colleagues provide evidence of the therapeutic potential of autologous nasal chondrocyte-derived cartilage grafts in repairing arthritis-damaged knee joints.

**Figure 1:**
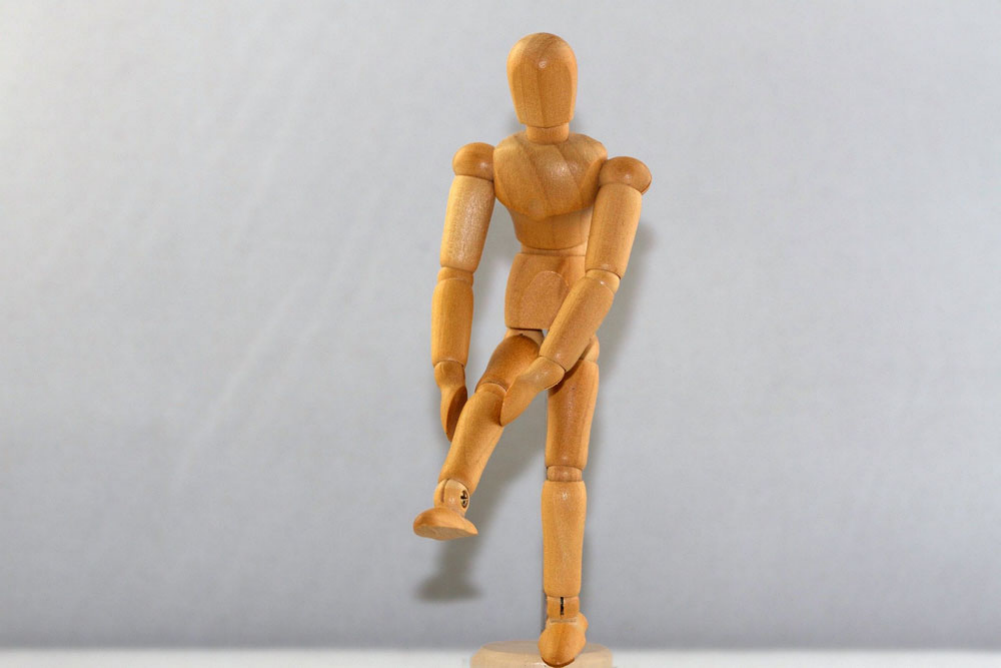
Pixabay

Osteoarthritis (OA) affects around ten million people in the UK and most commonly affects the knee. The progressive loss of articular cartilage that occurs within the joint is associated with significant pain, loss of mobility and reduced quality of life. Anti-inflammatory drugs are used to alleviate pain, with joint replacement reserved for end-stage disease due to the finite durability of the implanted prosthesis.

Recent years have seen sustained interest in developing regenerative cell-based therapies to repair damaged cartilage. One such therapy is autologous chondrocyte implantation (ACI), in which the patient’s own articular chondrocytes are harvested, expanded ex vivo and reinjected together with an extracellular matrix, such as collagen, in order to repair focal cartilage defects. However, the success of ACI in OA is limited by the inflammatory environment into which the cells must be implanted and the impaired quality of chondrocytes taken from diseased cartilage.

The healthy cartilage of the nose offers a potential alternative source of chondrocytes to engineer cartilage grafts. Building on their own previous phase I safety study in humans and an ongoing phase II efficacy trial, Acevedo Rua and colleagues now report preclinical and clinical findings on the therapeutic potential for autologous nasal chondrocyte-derived tissue-engineered cartilage grafts in treating osteoarthritic joints^1^.

In a series of experiments comparing grafts generated from either nasal or articular chondrocytes, the authors find that grafts derived from nasal cells maintain their cartilage-like properties to a greater extent when exposed to either a cocktail of inflammatory cytokines involved in OA or conditioned medium from osteoarthritic cells.

The authors demonstrate that nasal chondrocyte-derived cartilage grafts can successfully survive and engraft in human osteoarthritic tissue explants cultured in vitro or implanted into mice. They also engraft within the knee joints of sheep with OA and, after several months, appear to prevent further cartilage degeneration and inflammation.

In a pilot study in two patients with OA, aged 34 and 36 years, autologous nasal chondrocyte-derived grafts were generated and surgically implanted with no adverse effects. Tissue repair was observed on imaging and both patients reported reduced pain, improved joint function and improved quality of life.

While larger studies are ongoing, these data in animals and a small number of patients provide support for the use of nasal chondrocytes in tissue engineering approaches for osteoarthritis, potentially providing a way to mitigate the symptoms of this debilitating disease.
